# Selected Methods of Therapeutic Interactions With People With Mild Symptoms of Autism Spectrum Disorder

**DOI:** 10.3389/fpsyt.2022.942218

**Published:** 2022-07-15

**Authors:** Kopańska Marta, Chojdak-Łukasiewicz Justyna, Sochocka Marta, Leszek Jerzy, Podgórska-Bednarz Justyna, Banaś-Ząbczyk̨ Agnieszka, Ochojska Danuta

**Affiliations:** ^1^Department of Pathophysiology, Institute of Medical Sciences, Medical College of Rzeszow University, Rzeszow, Poland; ^2^Department of Neurology, Wrocław Medical University, Wrocław, Poland; ^3^Hirszfeld Institute of Immunology and Experimental Therapy, Polish Academy of Sciences, Wrocław, Poland; ^4^Department of Psychiatry, Wrocław Medical University, Wrocław, Poland; ^5^Institute of Health Sciences, Medical College of Rzeszow University, Rzeszów, Poland; ^6^Department of Biology, Institute of Medical Sciences, Medical College of Rzeszów University, Rzeszów, Poland; ^7^Department of Psychology, Institute of Pedagogy, College of Social Sciences, University of Rzeszów, Rzeszów, Poland

**Keywords:** autism spectrum disorder, therapeutic methods, biofeedback, symptoms, patients

## Abstract

In this review we present the behavioral aspects of interaction in people with autism spectrum disorders (ASD), taking into account some aspects of pharmacotherapy. In the treatment of people with ASD, an individual approach to emotional, social and cognitive functioning is very important. The specificity of symptoms and their severity in people with ASD results from deficits/disfunction of various areas of the brain and is associated with different levels of intelligence. This manuscript considers selected methods of interaction with ASD patients with normal IQ. Due to the different ways of functioning, these people often find it difficult to adapt to social expectations. The most important thing is to understand their perception of themselves and the world around them in order to support them in coping with the daily challenges. Due to the increasing problem, more and more attention is being focused on early detection of ASD, what allows to intervene as fast as possible and in consequence affect the quality of life of people with this dysfunctions. However, participants with mild autism symptoms are still difficult to diagnose in the practice. The effectiveness of the therapy depends largely on the cooperation of educational institutions. It is also necessary to contact specialist clinics, including a mental health counseling center. However, in the case of children and adolescents, the cooperation between the therapist and their parents is the basis. Systemic family therapy is also important in adults with ASD. An overview of the methods of therapeutic interactions in ASD, what may be helpful in diagnosing of mild ASD, were presented in our manuscript.

## Introduction

Autism spectrum disorder (ASD) is a neurodevelopmental disorder characterized by deficits in social communication and social interaction in many contexts with the presence of limited interests and repetitive behaviors (1, 2). In the case of people with ASD, the sooner specific therapeutic actions are taken, the greater the chances for better functioning in adulthood. Children with mild symptoms of ASD, with normal or higher intelligence, are usually diagnosed later, after the age of three, due to their low expression in early childhood. Their behavior is in many respects the same as their peers, hence the differential diagnosis can be difficult. These children reveal great difficulties in contacts with other children, it is difficult for them to empathize with the emotional states of other people and understand their intentions, as well as to adapt to changing environmental conditions, hence they are often referred to as socially maladjusted children. However, despite a slightly different way of functioning, they can adapt to specific situations and meet social expectations. In addition, they often have a chance to stand out above the average thanks to their specific abilities (1, 2), and consistency in pursuing their interests. As part of the impact on children with ASD, it is necessary to adapt children’s education to their needs, set goals focused on developing the necessary skills. The main environment influencing a child’s development is the family, which is why it is very important to support it properly. On the one hand, it is necessary to work on the entire family system, improve relations in the family, pay attention to the roles performed in the family, so as not to burden individual family members, especially siblings, and at the same time create circumstances conducive to adapting to the situation and organizing time, especially free time, so that all family members can discharge their emotions and ensure a proper quality of life. The second important, and very important social space is school. Observation of the child by teachers and the appropriate approach to the disciple with disabilities is crucial in his improvement. Most of the authors underlines that early diagnosis and interventions, such as in the pre-school period or earlier, can contribute to tremendous progress in terms of symptoms and skills thereafter (3). Children with mild ASD, having IQ within the normal range, usually attend public schools. Nevertheless, they require support from teachers and therapists. It is not easy for them to be in the world around them and understand the standards prevailing in it, and create appropriate interactions with their peers. Effective therapy is associated with the selection of appropriate forms of therapeutic interactions. First of all, it is essential to create an appropriate therapeutic relationship, which is associated with several mutually interacting factors. Working with children with ASD requires exceptional patients and often constant experimentation on the side of the therapist how to reach their inner world. On the one hand, tangible benefit are brought by directive forms of therapy, that include elements of behavioral analysis, or in the case of young children, various forms of forcing contact (derived from holding therapy) (4, 5). On the other hand, it is worth using as a supplement non-directive methods of interactions (including animal therapy, sensory integration, behavioral modeling). Noteworthy are also modern solutions that do not yet constitute therapies with proven effectiveness, although the results of the pilot studies are very promising, such as the use of “Short-Term Deep-Pressure Portable Seat” on behavioral and biological stress in children with ASDs (4), but they will not be the topic discusses by presented article. Regardless of the type of interactions, the therapist must guide the therapy process, focus on explaining the mechanisms of incorrect behavior, and finally support in shaping adequate ways of functioning, as well as strive to eliminate inappropriate habits. It is necessary to analyze the signals coming from the child in various everyday situations. The therapist’s task is to explain to parents the assumptions of different forms of therapy, stages of therapeutic work, and a joint analysis of the child’s undesirable behavior in various situations, specially interactions that may be incomprehensible to parents. It is necessary to accept the individuality of each child and develop an individual program of interactions. The presentation made in this article will be important for caregivers/parents of people with mild symptoms of ASD, who get lost in a plethora of various therapies, including those with scientifically undocumented effectiveness. The article is therefore of a practical nature, presenting a compilation of selected reports from the last 5 years.

## Materials and Methods

The purpose of this review was to identify selected methods of interactions with people with ASD (with IQ within normal range) described in the literature. Seeking the appropriate interventions can be an overwhelming and frustrating experience for parents. The primary search strategy was conducted using the following keywords: “autism” OR “autism spectrum disorders”’ AND “therapeutic methods.” Studies published in English and Polish languages were considered.

**FIGURE 1:**
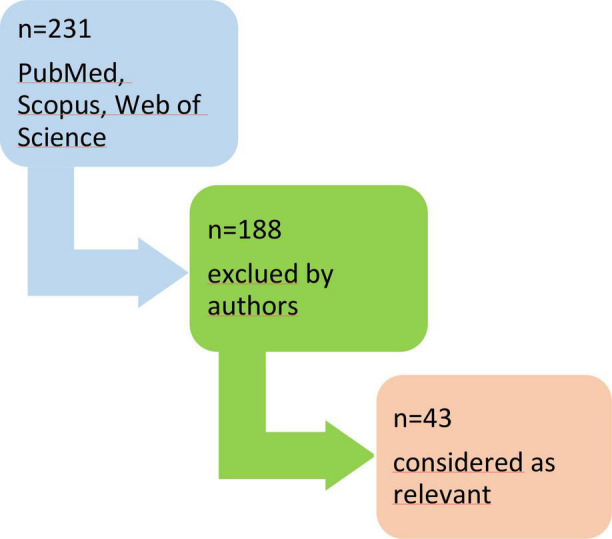
The process of data collection.

At the end of the selection process, 43 studies were included in this review. A simple chart of a selected publication is shown in Figure 1.

The authors of the article cited only selected items from the list of qualified publications on therapeutic interactions for people with ASD, due to the limited framework of the article. However, the most frequently repeated and relevant aspects in this field was taken into account. To the research items mainly from recent years have been selected (from 2015), which related to the most frequently used methods of therapy. Some articles from 2006 to 2014, which focused in detail on the effectiveness and description of the therapeutic interactions, were also included. An additional criteria for include these articles in the review was the analysis of the therapy impact on people with ASD with the correct level of intelligence. The main rejection criteria for articles were repetition of manuscripts and pharmacological aspects of ASDs.

## Results

**FIGURE 2:**
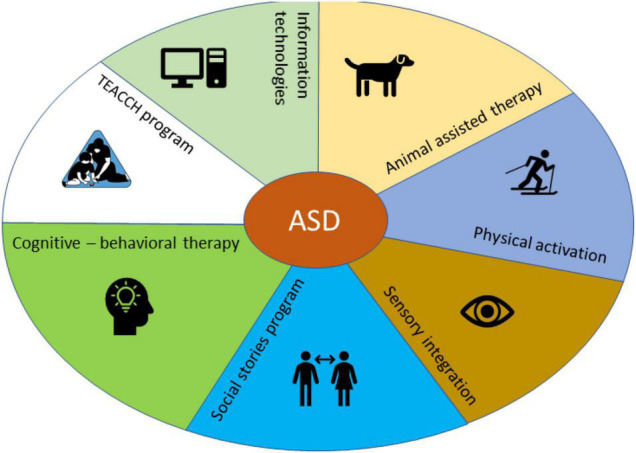
The therapeutic interactions for patients with ADS.

In this review the authors present various forms of behavior modification for patients with ASD such as: TEACCH program, cognitive—behavioral therapy, social storytelling program, Sensory Integration, physical activation, animal-assisted therapy and information technology, as those most commonly discussed in the selected material. The selected methods are approaches that are either highly therapeutically effective for autism or are frequently used in the treatment of autism, and are therefore listed in Figure 2.

### TEACCH Program

**FIGURE 3:**
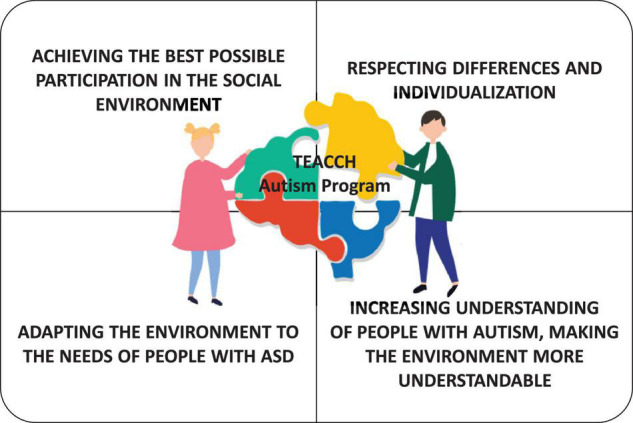
The main aspects of TEACCH—Autism program in developing the independence of the person with ASD. Graphic from https://pixabay.com.

The visible effects of work with children with ASD are brought about by the TEACCH program (Treatment and Education of children with autism and children with conjugated communication disorders—Treatment and Education of Autistic and related Communication—handicapped Children) (6, 7). Developed by Schopler and his colleagues in North Carolina (United States), this program has been in use for approximately twenty years. It focuses primarily on the therapy and education of children with ASD and multiple communication disorders. The task of TEACCH therapists is to develop individual intervention programs, as well as to cooperate with the school and other institutions. It is also important to take into account the role of parents, activate them through participation in training and individual meetings with the therapist, as well as to help in creating support groups. An individualized program addressed to a person with ASD is based on the analysis of its potential capabilities and competencies, as well as interests and takes into account specific difficulties. The main goal of the therapy is to develop the independence of the person with ASD at every stage of his/her life, therefore he/she should interact with different people, contact with peers is especially important here (Figure 3).

Adaptation to the requirements of the environment is involves, among others, the organization of the structure of the environment. It is necessary to properly organize the physical environment, establish an individual daily schedule, work system, and pose new challenges to generalize skills in changing conditions. Similar principles are observed in different environments, both in the classroom and at home. A lot of visual aids (inscriptions, pictures, photos, and films) are used during the class. In therapeutic work, it is necessary to formulate clear expectations and set realistic goals. Similar rules and standards must be followed at school and home. Taking into account the behavioral aspects of the interactions, it is necessary to strengthen the desired behavior, taking into account the child’s level of functioning and his interests. It is necessary to awaken motivation activated by an appropriate reward, issuing specific and clear commands, creating an action plan, presenting appropriate behavioral, and speech patterns, and learning the right reactions in social situations through observation of peers. In the therapeutic work, methods of extinguishing undesirable behaviors are also used, e.g., in the situation of revealing maladaptive behaviors, e.g., aggression toward others, the “Time-out without exclusion” technique may be useful (when the student receives a reward for a certain period of time after the undesirable behavior has occurred, but may remain in the place where this behavior occurred, in a situation when, e.g., he apologizes, we give him back the desired object or we allow to continue playing or performing previously commenced activities). In some situations, the “Time-out with exclusion” is necessary (withdrawal of positive reinforcements and temporary removal of the child from the environment in which he/she stayed). In turn, “Holding” is rarely used, e.g., in a situation where a disciple is completely out of control over his/her behavior and may harm himself/herself or others). At various times, “hyper-correction” is also necessary, when the disciple is obliged for the damage, and make amends in the event of inappropriate behaviors, e.g., cleaning. Another variant of hyper-correction is the cycling repetition of proper behavior (4). Especially in younger children, education related to the creation and consolidation of specific behavior patterns in the situation of interaction with another person is necessary, as well as practicing the skills of greeting, saying goodbye, thanking, and introducing into new situations. Many children with mild symptoms of ASD manage it, but in stressful situations they can activate unwanted auto-stimulating behaviors, such as flapping their hands, picking one’s nose, rocking, or running away from an undesirable place. People with mild symptoms of autism expect specific situations to be clarified, such as why one should raise their hand when the teacher asks a question, rather than the immediate answer, that one have to patiently wait for their turn, i.e., that is, follow group rules. On the other hand, caregivers like their peers, must have a certain amount of specific knowledge about the specifics of responding to people with autism. An example of this is the case of a student diagnosed with Asperger’s Syndrome (own experience of one of the authors of the article) who accused his parents (teachers) of hiding the diagnosis of ASD at school, which was why he was rejected by his peers, who often joked about him and invented various traps, difficulties (e.g., storing the necessary writing tools or hiding the hat in winter). This in turn caused a sense of helplessness and intensification of movement stereotypes. Similarly in college, until the fourth year, this young man was considered a “weirdo” and utterly rejected by others. It was only when, at the suggestion and presenting Temple Grandin, a film about an autistic professor of animal science, that he opened up to others, talked about his situation, and made others treat him differently and accept him. For many people with ASD, especially those with a normal IQs, changing their perception of themselves and the world is essential to successfully cope with a variety of situations. Not all perception patterns of other people can be broken due to difficulties in understanding the emotional or other person’s emotional states due to deficits in brain function. Nevertheless, various exercises and forms of therapy bring results.

### Cognitive-Behavioral Therapy

Nowadays, cognitive-behavioral therapy is most often used in working with people with ASD with low manifestation of autistic symptoms. Research indicates that it is common for adolescents and adults with Asperger’s syndrome to manifest a variety of disorders associated with autism, including increased anxiety, affective disorders, eating disorders, obsessive-compulsive disorder, and substance abuse (8, 9). They are usually characterized by a low level of tolerance to stress and impulsive reactions related to it (10). Such behaviors in turn, make it difficult to interact with other people and lead to lower self-esteem and a negative attitude toward oneself and the world. Cognitive-behavioral therapy aims to change misconceptions and reduce the cognitive deficit initiated by disturbances within the central nervous system. In addition, this therapy focuses on problems related to difficulties in perceiving and expressing emotions, as well as disorders in creating interactions with other people (11–14). As already mentioned, despite the correct level of intelligence of people with Asperger Syndrome, highly functioning individuals have problems with understanding non-verbal behavior, with metaphors and intuitive behavior. Therefore, it is necessary to practice the ability to recognize other people’s states, ask about their feelings and develop negotiation skills, and control in expressing negative assessments (e.g., a student with ASD with a very sensitive sense of smell perfectly creates various fragrances compositions, but also often tells the teachers that they “stink”). Exercises in the skills of analyzing the behavior of other people make it easier to interact with them. Therefore, as already mentioned, it is necessary to learn the principles that should be followed by a person with ASD, making new acquaintances and sustaining them. It is also necessary to learn how to resolve conflicts effectively. The development of such skills would also be useful for many people who have not been diagnosed with ASD.

### The Social Stories Program

For people with ASD, a specialized program called “social stories” is also proposed (15). It consists in presenting imaginary stories related to a specific social situation, to which a person must react appropriately. It is often necessary to repeat certain scenes several times in order to establish the behavior pattern at such moments. Such an exercise is very effective in developing the ability to understand the situation and apply the appropriate response. The educational behavior of relatives, in particular parents toward their own children, also plays a key role. Modeling the correct behavior or explaining the rules during play can contribute to the improvement of the child’s functioning in the community, i.e., peers. Emotional education is also beneficial. During this therapy, the patient draws knowledge about various sources of emotions, methods of recognizing them, and ways of expressing them, as well as the role of emotions in human social life. Therefore, the key here is to consolidate patterns of behavior of how to respond to the emotional states of others.

For this purpose, various scales may be useful to describe a given emotion, for example, in a given image, by using numerical values. The patient may also be asked to act the behavior assigned to a given emotion or to present it by modulating facial expressions. The above exercises facilitate the correct interpretation of the interlocutor’s emotional states, but also develop the ability to express their emotions so that they are better understood by the social environment. This therapy helps in modifying self-image, positive thinking about oneself, and perceiving oneself as a valuable person. Conducting the internal motivational dialogue is also helpful in maintaining such an image. This dialogue should include phrases, such as: “I am doing great” or “I am a valuable person” (14). An important element of this therapy is learning to control the outbursts of anger. The therapist is to help in the implementation of the assigned action strategies. The therapist should motivate a given person to analyze the problem before an outburst of anger, to consider possible solutions and choose the most appropriate one, and then to assimilate the task by playing a role. In addition, the embodiment of relaxation in such stressful circumstances for the patient will help to control the fear of various unexpected situations. The right tactic of action teaches the patient that he is completely responsible for the course of the next situation, which motivates him to modify his behavior. The use of many exercises teaching new skills raises the conviction of their strength and thus facilitates establishing healthy relationships with the environment in which a given person resides. Cognitive-behavioral therapy improves the overall quality of the patient’s life by consolidating behavior adequate to the situation and becoming aware of the determinants of various difficulties in everyday life. In many cases, the therapeutic effects are brought by the lack of focusing on the mistakes and failures of a person with ASD, and focusing on mobilization to action. One should also pay attention to own behavior in order not to discourage a child or adult with ASD from working. If the patient misunderstands the instructions, it is important to repeat them, paying attention to the friendly and pleasant tone of speech. It should also be remembered that if the patient does not understand what to do, help him again by giving a clear indication, e.g., bringing the object closer than intended or presenting an action plan consisting of a sequence of images or verbal cues, to assist in the implementation of specific operations. Certainly, alternative forms of therapy are worth using while working with people with ASD, although many specialists, among others, within the Applied Behavior Analysis, assume that the implementation of a particular method of interaction excludes the implementation of another. The leading therapy is optimal, however, considering the various supportive effects.

### Sensory Integration

**FIGURE 4:**
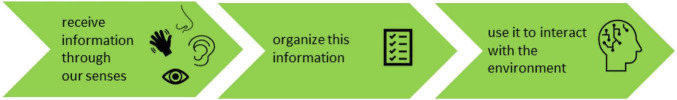
Main aspects of sensory integration.

In the case of younger children with ASD, sensory integration therapy brings significant effects (16). It turns out that especially people with autism have difficulties in the overall perception of specific stimuli due to the different levels of sensitivity of different senses. It happens that a child with this problem can hear particular sounds perfectly, and others do not reach him, e.g., he can hear the buzzing of a distant fly, and does not perceive the sounds of human speech. On the other hand, he reveals hypersensitivity to smells and does not feel different parts of the body. Usually, the lack of coordination among many children in receiving information from different senses simultaneously triggers a feeling of confusion. If specific information from different receptors with specific stimuli is transmitted in an organized, integrated way, the brain processes it properly, which in turn promotes the development of perceptual processes as well as learning and shaping behaviors. Therefore, an important goal of this therapy is to stimulate and inhibit specific reactions to stimuli, and to develop tolerance to external signals (the therapist controls their severity and frequency) (Figure 4). Before a therapist offers the child an exercise, he should first perform it on himself, measuring his reactions under the influence of stretching, pressure, or striking certain objects. His sensitivity to feedback from a child is essential here. In a situation of hypersensitivity to given stimuli, it is necessary to inhibit the protective reflex against contact with a given object. For example, in the situation of hypersensitivity to touch, it is necessary to gradually increase the strength of a given stimulus by pressing delicate materials such as a feather on the hand, and then acting more and more harder—with a pebble. In turn, in the case of sensitivities to touch, the therapist should start with stronger, to more delicate ones. Often an indicator of the lack of sensitivity of various parts of the body are the statements of people with ASD, but also auto-aggressive behavior, such as severe scratching of the “insensitive finger.” It is useful here, among others, deep massage, submerging hands alternately in warm and cold water. Similarly, in case of olfactory hypersensitivity, first avoid fragrances and gradually introduce the smell. When a child is hypersensitive to flavors, start with mild food, through sour and finally bitter food. Hearing hypersensitivity, in turn, requires the suppression of various sounds, whispering, and gradually amplifying sounds. In the case of visual hypersensitivity, it is necessary to avoid glowing objects and strong light at the very beginning and slowly get used to the stronger visual stimuli. Hypersensitivity to the above-mentioned receptors, in turn, requires the use of stronger stimuli, and then a gradual reduction of their intensity. When working with younger children with ASD, it is important to use methods that focus on contact with the body. Many children with ASD reveal difficulties interacting indirectly with another person. Meanwhile, hugging, and direct, close contact ensure that the need for security is met. Often, children with ASD seek for alternative forms of closeness, hiding in small spaces, e.g., under a wardrobe or in a box. The sensations of touch and movement are conducive to the development of hand-eye coordination, deepening information about the body, which in turn favors the overall development of the child.

### Physical Activation

Children with ASD usually show motor dysfunction manifested by imbalance, difficulties in limiting the movements, as well as in the presence of co-movements and involuntary movements. In these children, often occur mobility impairments, including gait or the ability to participate in team games, such as football. During these activities, it can be noticed some kind of stiffness in the whole body and unusual coordination of movements. People with ASD, also high-functioning ones, have difficulties with performing alternative movements of the upper limbs, as well as in initiating movement at the right moment and problems with controlling the movement force, its speed, and overall movement planning. The motor disability of children with ASD contribute to greater problems in performing various everyday activities such as dressing or undressing, eating, holding objects, moving various objects, or writing.

**FIGURE 5:**
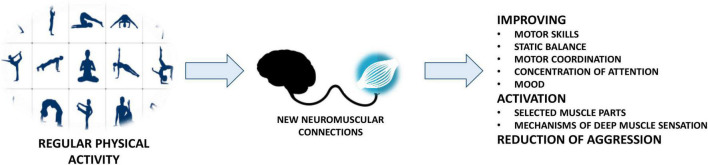
The role of physical activity in ASD therapy.

Therapy with the use of physical activity is crucial for the development of every person, not only suffering from dysfunctions but also healthy ones (Figure 5).

One can quote here a very popular and convincing statement that “the movement can replace almost every drug, but all drugs taken together cannot replace the movement” (17). The development of motor activity in children with autistic features undoubtedly contributes to the improvement of cognitive and motor development indicators, which is particularly necessary due to the reluctance to undertake physical activity, or even avoid sports activity by these children (18). Physical exercises mainly improve motor skills, such as balance and jumping, but also dexterity, locomotion, and throwing. In addition, physical recreation improves static balance in people with ASD, which is also associated with the improvement of motor coordination, which affects the quality of spatial and temporal multidimensional movements. To analyze this factor, a strain guage platform coupled to a computer by means of a suitable program for example, Mathcard. Based on existing studies, it can be concluded that if the amplitudes are disturbed beyond the norm before the exercise, then in the case of the state after activity, the amplitude approaches the x and y axes, which is reflected in the decrease in amplitude characteristic of healthy people. However, for the results of the exercise to be permanent, attention should be paid to the regularity of exercises and a long-term lasting, minimum of three months therapy (18). Only then there will be a positive effect on changes in the movement sphere in people with autism. The aim of motor therapy is therefore to stimulate the brain to acquire new neuromuscular connections. Regularly repeated physical exercises, in addition to activating selected muscle parts, activate the mechanisms of deep muscle sensation, develop concentration of attention, improve the mood of a child or an adult, promote rebound tension and reduce the level of aggression, and at the same time promote autonomy in everyday activities.

### Animal-Assisted Therapy

**FIGURE 6:**
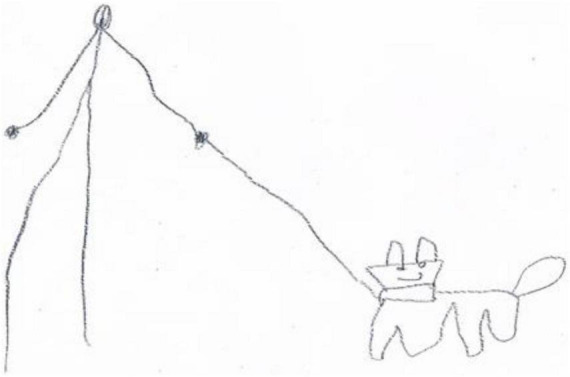
A woman who goes for a walk with her dog—Jaś, 9 years old.

Research and observations also confirm that animal therapy, otherwise known as zootherapy, plays an important role. Neuro-mapping of the brain and the statements by people with ASD indicate that they have difficulty perceiving the human face, and are much more focused on animals. Therefore, other areas of the brain are responsible for the representation of human and animal faces (19). This is also illustrated in Figure 6, the drawing of a 9-year-old boy with the normal level of intelligence, with ASD. The boy referred to the topic suggested by one of the authors of the article: “Draw a woman who goes for a walk with her dog.” It turned out that Jaś presented the dog quite accurately, and the woman very generally and schematically, without a face. In drawings on other topics, the boy also drew human faces without many expressions.

Therapists rightly emphasize that, on the one hand, interactions with animals do not require reciprocity in interactions, besides, animals do not exert pressure on people, and enable them to experience positive emotions (it has been proven that the very presence of animals significantly improves mood) also improves sensory integration thanks to close physical contact (many people with ASD have difficulty in direct physical contact with another person, and hugging a flat surface or animals evokes positive emotions). The presence of animals for patients can be not only a source of relaxation but also motivation for patients. The analyses show that children with ASD, thanks to contact with animals, reveal a greater willingness to play, increased concentration, and responded better to the environment (20). Therefore, communication with animals improves the quality of social contact. The conducted analyses show that the creation of strong bonds with animals by people, especially with Asperger’s syndrome, may result from their specific way of perceiving stimuli. This is because both animals and people with autism perceive the world through images and create an individual perception of the world by combining details and small particulars.

Moreover, people with ASD who find it difficult to create relationships with other people may find loyal companions in animals. Animals do not pay attention to the way of communication, incorrect termination of conversations, inadequate response, misunderstanding of metaphors, irony, or jokes. This makes these patients feel safe and comfortable when staying among animals. Therefore, the participation of animals in the life of people with ASD usually improves their quality of life. A variety of animal species can be used for zootherapy, especially dogs, dolphins, or cats. Dogs are considered the closest species to human. It is claimed that just like a human, they react to specific situations since they have been cooperating with people for thousands of years. There are many similarities between the functioning of the dog’s brain and the human brain. The research show that dog therapy not only affects children’s wellbeing and positively influences cognitive processes, but also supports, for example, learning to read, or stimulates the expression of complex statements (20, 21). The use of feline therapy, i.e., cat therapy, contributes to calming down and silencing the patient because cat purring has a very relaxing effect on the human nervous system. In addition, the presence of a cat affects the building of correct social relationships. Researchers emphasize that often feline therapy is more desirable comparing to dog therapy because the dog’s noisy behavior and its rapid movements may be too strong stimuli for people with autism, besides its smell is more intense compared to the neutral smell of the cat (22). Also, the use of hippotherapy involving not only horse riding, but mainly horse care, cleaning or feeding, affects the wellbeing and increases self-confidence and self-esteem. This is mainly due to the fact that people with dysfunctions realize that they are able to influence the movement and activities of large animals. Equestrianism brings benefits in the improvement of motor functions, especially in the case of maintaining static balance, which is associated with specific pelvic work in the saddle that moves forward and backward, as well as rotational movements. Contact with dolphins, however, stimulates intellectual functions. The sounds emitted during the echolocation of dolphins through ultrasound waves stimulate the brain and support the process of speech development. However, further research is necessary to confirm the effectiveness of this form of therapy (23, 24). Certainly, animal therapy does not cure ASD, but it favors positive changes in the functioning of people with ASD, improves cognitive processes, and contributes to better social adaptation. Zootherapy also reduces anxiety and helps to reduce the feeling of loneliness.

### Information Technologies

Research confirms that today the mass media play a huge role in creating the image of people with mild autism and Asperger’s syndrome. Often people who reveal autistic features with an appropriate level of intelligence become very critical of themselves, feel stigmatized, because people around them often avoid contact with them, and treat them as weirdos. They tend to find it harder to understand that their specific reactions in different situations create anxiety and fears in others that interfere with their interactions. Therefore, it is important to disseminate knowledge about otherness and its determinants. Positive creation of the image of people with autism contributes to the improvement of their quality of life. New media is an appropriate means of building a specific narrative about this dysfunction, while this process may not be feasible or difficult in the real world. The mass media are thus a way to create their own identity, i.e., a set of beliefs about themselves (25, 26). Blogs are the optimal space for people with autistic features. It is easier for them to communicate anonymously with others about how they feel and the way they are. Indirect interactions, they find it harder to enter into relationships and understand the group’s behavior. Sometimes, too much noise is enough to make a person with ASD feel so lost that they are unable to comment ion the topic. In addition, the need for auto-stimulation to cope with tension (e.g., turning around, swaying) increases. This secondarily intensifies the negative reaction of the group and may intensify the feeling of being stigmatized. Blogs have various functions: educational, informative, or entertaining. Thanks to blogs, people with Asperger’s syndrome can learn to communicate more efficiently. It is often said that if other people find out about a given person, then that person begins to exist. This conviction motivates the acquisition of identity through electronic media. People with ASD with an appropriate level of intelligence are characterized by a hidden disability, which, however, can be revealed in the form of uncontrolled behavior at any time. Through the mass media, these people learn to manage their identity and decide on the procedures for concealing negative traits, in order to be accepted in the social space. This, in turn, facilitates coping in everyday situations. Blogs are a kind of autobiographical material because they describe social situations, as well as the attitudes and actions of the author toward these situations. Many researchers using their analysis of blogs of people with this dysfunction, draw attention to the type of topics, language style, as well as the topics discussed. Thanks to such an analysis, it is possible to get to know these people, and their experiences, and thus to study their identity. On blogs of people with Asperger’s syndrome, one can often see negative emotions related to the process of self-stigmatization, that is, the process of feeling and responding to difficult situations occurring in social relationships, and showing positive or neutral elements related to the auto-identification process, identifying with the process of coping with problems. Thanks to blogs, people with autism are able to find their subjectivity among neurotypical individuals, although the blogger’s entries often contain statements that being among other people is associated with the need to pretend the behavior of other people, and not being themselves. Nevertheless, electronic diaries are an environment that gives a great sense of security due to the lack of direct interaction with other people. A positive factor is thus the lack of pressure and the simultaneous possibility of interactivity while creating one’s own subjectivity in the virtual world. The media, as a supportive measure have a positive impact on interpersonal contacts and participation in social life, which is of great importance in gaining self-confidence and raising self-esteem. Summarizing, the Internet is a way to become independent, express one’s own beliefs, influence the acquisition and practice of newly acquired skills, or the opportunity to share interests and abilities (27). Numerous blogs are also run by parents who have contact with the autism daily. Their role in the virtual world is huge because they create the right vision of children with ASD without any stereotypical associations typical for people without knowledge about this dysfunction.

Considering the neurobiological conditions of the spectrum of autistic disorders, various methods of brain stimulation are appreciated more and more. These include neurofeedback. Neurofeedback, or brain-computer interface (BCI), is a therapy related to obtaining feedback signals in the visual or acoustic form, showing the changes in the physiological condition of the body. Neurofeedback is a non-invasive method of monitoring the brain’s bioelectrical activity through EEG signal analysis. Neurofeedback training is an auxiliary measure for observing the patterns of brain waves, which are closely related to specific brain conditions, the patient’s mentality, and his appropriate behavior. Neurofeedback uses slow—theta and fast waves—beta. This is related to the creation of a stable concentration and activation of the brain capable of repairing processes. The patient can influence the flow of certain brain waves by performing specific tasks and evoking a given emotional state in oneself. The therapist and the patient himself have the opportunity of real time observations of brain waves on the monitor, which constitute a feedback. Mobilizing mentally, the patient can contribute to the development of self-regulation of his mental state. The method applied in neurofeedback is based on Pavlov’s experiments, taking into consideration the similarity of the mechanisms of action. Thanks to the use of neurofeedback therapy, it is possible to accelerate the learning process, optimize attention processes, as well as eliminate the level of stress, anxiety, or impulsiveness. In addition, this therapy has a positive impact on the development of brain relaxation skills, raising the level of vigilance, controlling the state of mind, controlling concentration by using mainly beta waves, as well as exercising mindful functioning by controlling impulsivity. This therapy thus increases the brain capacity and eliminates numerous symptoms related to autistic behavior. It has been proven that the discussed type of therapy brings many satisfying effects, such as increasing memory resources, improving the processes related to effective association, improvement of visual and motor coordination, higher level of concentration, development of concentration, improvement of concentration, improvement of emotional control, as well as increased level of self-assessment and motivation for undertaking any action. According to the EEG, about 83% of people with Asperger’s syndrome have lower cerebral activity in the region of the right temporal section compared to the homological areas in the left hemisphere. The use of neurofeedback, therefore, has a normative effect on the defective area of the brain. Knezevic et al. in the pilot project studies, performed on the nineteen consecutive clients (16 male and 3 female) reported that 40 neurobiofeedback sessions (NFB was introduced as a computer game in which one could obtain points by maintaining a focused and relaxed mental state as indicated by brainwave patterns) coupled with the training of metacognitive strategies have a positive effect on executive functioning in clients with Asperger’s syndrome including their planning efficiency, speed, and the ability to switch sets and inhibit certain responses (28). An interesting variation of neurofeedback therapy is the use of an innovative method in the form of a “Brain-computer” game that supports communication between the brain and the computer (29–32).

In summary, the application of neurofeedback causes the normalization of the brain regions possibly related to the behavioral, cognitive, and emotional functions. Neurofeedback therapy in the form of using games is quite effective, especially for children, as it increases their curiosity and concentration, which motivates them to perform a greater number of tasks. In Friedrich and coworkers’ research (33), children (thirteen participants with ASD) took part in 16 sessions of based on a game neurofeedback therapy. They compared the standard method of enhancing mu to the bidirectional training of EEG mu suppression and enhancement (8–12 Hz over somatosensory cortex). The children learned to control mu rhythm with both methods and showed improvement by increased mu suppression, improved emotion recognition and spontaneous imitation, and also significantly better behavior in everyday life (which was reported by parents).

Participating in therapies for people with a higher degree of severity is difficult if not impossible due to necessary prerequisite behavioral, language, or cognitive skills. Solutions for less-functioning children are needed to participate in a greater number of tests and therapies. A novel teaching tool could be the Acoustical Guidance (TAGteach) (33). A new method based on the principles of standard treatment for autism (34, 35), and using conditioned auditory markers to shape complex behavioral sequences in successive approximations.

## Pharmacological Management of Children and Adolescents With Autism Spectrum Disorders

Medications are sometimes used to target symptoms experienced by some children with ASD. It is fully agreed that behavioral treatments remains the mainstay of treatment of the core symptoms of ASD including communication deficits, social interaction deficits, and repetitive behavior. However, adolescents with ASD may also have severe behavioral challenges, including irritability, aggression, and hyperactivity. Despite the high prevalence of psychopathology requiring pharmacological management, numerous medications are used to treat the ASD symptoms (4, 5). Treatment of ASD is challenging due to the variability of the clinical presentation of ASD and commonly occurring comorbidities. It should be noted that children with ASDs may not respond to medication in the same way as normally developing children. There are currently only two drugs, atypical antipsychotics (risperidone and aripiprazole), that are FDA approved for the treatment of ASD-related irritability disorders characterized by aggression, self-injurious behavior (SIB), and severe tantrums (36). Pharmacological treatment is used to treat ASD-associated symptoms, especially irritability, sleep disturbances, anxiety, depression, obsessive-compulsive disorder, and repetitive behaviors. Limited clinical data have supported treatment with SSRIs, CNS stimulants, or NMDA-receptor antagonists (Table 1). Because the important role of the neuroinflammatory factors mediated by glial cells, in the pathogenesis of ASD is also considered, it is proposed to introduce anti-neuroinflammatory therapy for ASD (37). Some potential directions of research include medications that modulate the metabolism of neurotransmitters such as glutamate, γ-aminobutyric acid, or block acetylcholinesterase. To date, research into the pharmacologic management of autism-related symptoms is limited by studies using less rigorous methodologies, including open-label trials with small sample sizes and heterogeneous patient populations across the autism spectrum.

**TABLE 1:** Medical management of ASD.

Symptoms	Medication
Irritability and aggression	Antipsychotic medications (risperidone, aripiprazole)
Mood, anxiety and repetitive behaviors	SSRI (citalopram, fluoxetine, fluvoxamine) Anticonvulsants (valproid acid, divalproex sodium) and lithium
Hyperactivity and inattention	Psychostimulants (methylphenidate), SNRIs (atomoxetine), α-adrenergic (guanfacine, clonidine)
Sleep disturbances	Melatonin, α-adrenergic (guanfacine, clonidine), trazodone
Behavioral disturbances	GABA-modulating agents (arbaclofen) NMDA-receptor agonist (ketamine) NMDA-receptor antagonist (memantine)

## Conclusion

People with ASD with the normal level of intelligence, thanks to the complementary forms of therapy, have a good chance for a much better social functioning and building healthy relations with other people. Fifth compares people with ASD to sirens, who are unable to communicate with the environment, and cannot thus present their true nature. It is also an entry from a person affected by Asperger’s syndrome: “Autism is not a disease but a different operating system in the brain. Most people use Windows, but that does not mean that computers with Linux are handicapped? Of course not. Linux, or Unix in general, may be difficult to use and strange to the average person, but they have many applications. For example, servers that perform much better than Windows intended for home computers. According to Bernard Crespi therapies can develop in two main ways: (a) community modeling (attempts to establish social interactions) and (b) socializing of patterns (“involves working from inanimate to animate, *via* the incorporation of metarepresentation, imagination and novelty into cognition elements of individuals) with autism” (38).

People with ASD, especially those with autism of relatively high general intelligence, are able to learn specific patterns of behavior in a given society. They can create relationships with other people and adapt to specific conditions, and their otherness—often exceptional abilities—give them a chance to be recognized by the environment. Acceptance of otherness is associated with social education, especially in the mass media (39). Despite the progress in disseminating information about ASD, many people are not aware of it. People with ASD themselves, through their presence on blogs, can give a chance to Internet users to get to know their originality. However, the basis of social adaptation of high-functioning people with ASD is self-acceptance and open conversation about their otherness (40). Family and early intervention involving various forms of therapy, are of great importance in shaping the self-image and social adaptation. The effectiveness of the effects depends on the cooperation of the entire multidisciplinary team, which includes doctors, biologists, therapists, psychologists, pedagogues, speech therapists, teachers, support groups, but above all, parents and a person with ASD (41). Interventions aimed at peer groups also deserve attention, as they complement and constitute a link between the effects developed during therapies defined as effective, and functioning in everyday life (42). Early interventions based on the therapeutic method can improve the functioning of people with ASD. Due to the complex problems faced by people with ASD, it is important to conduct further research on this subject. However, despite the considerable progress made in the field of knowledge about therapy in ASD, there are still many systemic and organizational problems that need to be solved in order to improve the diagnosis and therapy of autism (43, 44).

## Author Contributions

KM, OD, and B-ZA: conceptualization. KM, SM, and LJ: writing—original draft preparation. P-BJ and C-ŁJ: review and editing. All authors contributed to the article and approved the submitted version.

## Conflict of Interest

The authors declare that the research was conducted in the absence of any commercial or financial relationships that could be construed as a potential conflict of interest.

## Publisher’s Note

All claims expressed in this article are solely those of the authors and do not necessarily represent those of their affiliated organizations, or those of the publisher, the editors and the reviewers. Any product that may be evaluated in this article, or claim that may be made by its manufacturer, is not guaranteed or endorsed by the publisher.
